# Does Neoplastic Disease Negatively Affect Serum Antibody Titers Against Core Vaccines in Dogs?

**DOI:** 10.3390/vaccines14090822

**Published:** 2026-09-18

**Authors:** Paola Dall’Ara, Claudia Pollera, Daniela Invernizzi, Sara Tomassone, Joel Filipe

**Affiliations:** 1 Department of Veterinary Medicine and Animal Sciences, Università Degli Studi di Milano, Via dell’Università 6, 26900 Lodi, Italy; paola.dallara@unimi.it (P.D.); claudia.pollera@unimi.it (C.P.); 2 Centro Veterinario Torinese, Lungo Dora Colletta 147, 10153 Torino, Italy; dr.danielainvernizzi@gmail.com (D.I.); saretta85to@icloud.com (S.T.)

**Keywords:** dog, neoplasms, canine oncology, core vaccines, antibody titration, PATs, VacciCheck^®^

## Abstract

**Background/Objectives**: Neoplasia currently represents the primary cause of death in companion animals worldwide, with one in four dogs developing a tumor during their lifetime. In this scenario, evaluating the immune status against core vaccine-preventable diseases becomes of primary importance to ensure adequate protection in such clinically fragile patients. This study aimed to assess the presence of protective antibody titers (PATs) against core vaccine antigens—canine parvovirus type 2 (CPV-2), canine distemper virus (CDV), and canine adenovirus type 1 (CAdV-1)—in dogs diagnosed with neoplastic disease prior to receiving any antineoplastic therapy. **Methods**: A total of 120 client-owned, core-vaccinated dogs with various malignant neoplasms were evaluated using an in-clinic semiquantitative assay (VacciCheck^®^). **Results**: Overall, high rates of seroprotection were maintained across the study population, with PATs detected in 93.3% of dogs for CPV-2, 86.6% for CAdV-1, and 79.2% for CDV. High protection levels were consistently observed across different sex, age, and body size categories. Solid humoral memory was retained across all tumor types, although dogs with hemolymphopoietic malignancies (especially lymphomas) exhibited slightly lower protection rates (91.9% for CPV-2, 83.8% for CAdV-1, and 75.7% for CDV) compared to other histotypes. Time elapsed since last vaccination influenced CDV and CAdV-1 titers, whereas CPV-2 immunity remained remarkably persistent even beyond 3 years post-vaccination. **Conclusions**: These findings indicate that high seroprotection against core vaccine antigens is maintained in dogs with neoplastic disease prior to antineoplastic therapy, with no clear evidence of major humoral impairment compared to historical healthy reference data, thereby reassuring veterinary clinicians and pet owners that routine core vaccine boosters may not represent an immediate priority in canine cancer patients.

## 1. Introduction

In recent years, advancements in diagnostic approaches, therapeutic options, and a greater emphasis on pet lifestyle have significantly extended the life expectancy of companion animals [1,2,3,4,5]. This longevity, however, has been accompanied by a rising incidence of different diseases, particularly neoplasms, in the canine population.

Managing the oncology patient presents multifaceted challenges for the veterinary surgeon, with the primary goal of maintaining the best possible quality of life. In this context, the strategic planning of prophylaxis against major infectious diseases is paramount. Currently, the field of immuno-oncology is garnering significant interest, not only regarding its role in anti-tumor surveillance, but also its therapeutic potential. Neoplastic diseases can induce profound immunological alterations, but there are still many aspects to be clarified, particularly the impact of different tumor types on the immune system. It is therefore imperative to determine if vaccine protective immunity is preserved despite the potential for cancer-related immunomodulation.

The entire scientific community, and in particular the main guidelines for dog and cat vaccination [6,7,8,9,10], classify pet vaccines as core and non-core.

Core vaccinations represent the cornerstone of preventive canine medicine, providing essential protection against highly contagious and even life-threatening pathogens, such as canine parvovirus type 2 (CPV-2), canine distemper virus (CDV), and canine adenovirus type 1 (CAdV-1, etiological agent of infectious canine hepatitis) [6,10,11,12]. All of these are modified live vaccines (MLVs) and, as such, are particularly successful in stimulating effective and long-lasting immunity. The presence and intensity of this specific immunity can now be easily measured using practical rapid in-clinics tests.

Although the 2024 WSAVA guidelines [6] now classify the Leptospira vaccine as core, all available formulations are inactivated and lack a direct correlation between humoral immunity and protection [11,13,14]. Consequently, these new core vaccines lie outside the scope of this work.

Beyond conferring individual protection, core vaccines ensure a pivotal herd immunity, thereby reducing the risk of community-wide outbreaks. Their universal adoption as a standard of care has resulted in a drastic worldwide reduction in the prevalence of these diseases within canine populations for over a century [6,10,12,15]. Vaccination protocols start in puppyhood and include booster shots during the adult life. The recommendations of the main international veterinary associations specialized in pet clinics, first of all World Small Animal Veterinary Association (WSAVA) [6] and American Animal Hospital Association (AAHA) [10], emphasize the need to optimize protection with individualized vaccination protocols considering specific factors like age, lifestyle, health status, and possible exposure risk to different pathogens, as well as owner attitude and socioeconomic constraints [6,10,16,17,18,19,20]. For example, older animals are likely to have lower vaccine-induced immunity and suboptimal health status including the development of different types of neoplasms and must be monitored closely; consequently, vaccination protocols may need to be modified, especially in geriatric patients [3,6,20,21].

Neoplastic diseases can diminish immune competence, and certain cancers and/or their treatments may diminish vaccine effectiveness by altering immune cell function [11,22]. This highlights the utility of pre-vaccination health status evaluation, especially in oncology patients.

Immunologically, core vaccines induce both humoral and cell-mediated immunity. They produce long-lived memory T and B lymphocytes and specific immunoglobulin responses that result in prolonged protection [5,11,15,23]. Persistence of protective antibody titers has been documented in different canine populations [6,11,24,25], and persistence of this feature is particularly significant in immunocompromised individuals, including patients with cancer undergoing treatment [26,27].

It is therefore crucial to understand the impact of malignant neoplasia on the immune system and whether standard vaccination protocols remain effective in such compromised hosts.

Therefore, the aim of the present study was to evaluate serum antibody titers against core vaccine antigens—canine parvovirus type 2 (CPV-2), canine distemper virus (CDV), and canine adenovirus type 1 (CAdV-1)—in dogs diagnosed with various neoplastic diseases prior to antineoplastic treatment. Furthermore, we sought to investigate the potential influence of tumor histotype, as well as host-related factors such as age, sex and reproductive status, body size, and vaccination history, on maintained protective humoral immunity.

## 2. Materials and Methods

### 2.1. Study Population and Study Protocol

Serum and plasma samples used for this study were collected over a two-year period (January 2023 to December 2024) both to analyze antibody titers and to monitor the overall health status of oncology patients. According to the Ethical Committee decision of the University of Milan, residual aliquots of samples collected under informed consent of the owners can be used for research purposes without any additional formal request of authorization (EC decision 29 October 2012, renewed with the protocol n. 02-2016). A total of 120 client-owned dogs presenting with malignant neoplasia, confirmed through clinical and pathological evaluations, were enrolled in this study. Overall, all dogs were in fair-to-good general health at the time of sampling. None of the dogs had received prior anticancer therapy except for surgical resection of the primary tumor, which also allowed for histopathological diagnosis. Neoplasms were then classified into five different categories based on their histopathological origin (see below). At the time of recruitment, all dogs had already been vaccinated with core vaccines. For comparative purposes, patients were stratified by sex and reproductive status (intact and spayed females, intact and castrated males), age (adult, senior, and geriatric, following the rule that smaller dogs have longer lifespans than larger dogs and vice versa [28,29]), breed size based on breed standards (small: <10 kg; medium ≥10–<25 kg; large: ≥25 kg), tumor histogenetic origin (hemolymphopoietic, mesenchymal, non-lymphomatous round cell, epithelial, and other types), and time elapsed since the last vaccination (<1 year, ≥1 year–<3 years, ≥3 years).

### 2.2. Antibody Titer Evaluation by VacciCheck^®^ 

Serological evaluation of protective immunity against canine parvovirus type 2 (CPV-2), canine distemper virus (CDV), and canine adenovirus type 1 (CAdV-1) was performed using the in-practice test VacciCheck^®^ Canine (Biogal, Kibbutz Galed, Israel, supplied in Italy by Agrolabo, Scarmagno, Italy). This rapid, semi-quantitative enzyme-linked immunosorbent assay (dot-ELISA) enables visual determination of IgG antibody titers against core vaccine antigens. The assay was carried out according to the manufacturer’s instructions, using combs with teeth pre-coated with antigens for all target pathogens. Serum/plasma samples as well as all combs and plates were brought to room temperature prior to testing and processed following the standard testing sequence.

The intensity of the chromogenic reaction was visually compared against a reference color scale (Comb Scale) provided by the kit, representing S-units from 0 to 6. The manufacturer has validated this scale correlating S values with conventional antibody titers: S0 corresponds to titers <1:20 for CPV-2, <1:8 for CDV, and <1:4 for CAdV-1, while an S3 value represents the threshold of serological protection (1:80 for CPV-2, 1:32 for CDV, and 1:16 for CAdV-1, respectively).

Antibody titers ≥ S3 were considered indicative of protective immunity; accordingly, dogs meeting this threshold were classified as having protective antibody titers (PATs, Table A1) [6,11,12,19].

### 2.3. Statistical Analysis

Statistical analysis was performed using GraphPad Prism 9 (La Jolla, CA, USA), with statistical significance set at *p* < 0.05. The chi-square (χ2) test was used to identify relevant differences between protected and unprotected dogs. Antibody titer results were normalized using log2. Shapiro–Wilk test was used to validate the normal distribution of the data, and non-parametric Kruskal–Wallis and Mann–Whitney tests were also used.

## 3. Results

### 3.1. Dog Population

A total of 120 canine serum/plasma samples were analyzed. Of these, 66 (55.0%) were females (22 sexually intact and 44 spayed) and 54 (45.0%) were males (35 sexually intact and 19 castrated). Collectively, 57 dogs (47.5%) were entire while 63 (52.5%) were neutered.

As expected, none of the subjects analyzed were under one year of age; therefore, the “puppy” category is not represented in this study. The dogs were 17 adults (14.2%), 78 seniors (65.0), and 25 geriatrics (20.8). Considering the breed, 69 dogs were of purebred (57.5%), while 51 were crossbred (42.5%), and the most representative breeds were Golden Retriever (9, 13.0%), followed by Beagle and French Bulldog (5 each, 7.2%), and by Border Collie, Boxer, German Shepherd and Pinscher (4 each, 5.8%). Considering size, 27 were small (22.5%), 39 medium (32.5%), and 54 large (45.0%). All dogs were oncology patients, and in particular 37 (30.8%) had a hemolymphopoietic neoplasm, 26 (21.7%) mesenchymal neoplasms, 23 (19.1%) non-lymphomatous round cell neoplasms, 20 (16.7%) epithelial neoplasms, and 14 (11.7%) neoplasms of other types (Table 1). Finally, all dogs were vaccinated at least once in their life with core vaccines before sample collection, particularly 43 (35.8%) < 1 year before sampling, 56 (46.7%) ≥1–<3 years before, and 21 (17.5%) ≥ 3 years earlier.

### 3.2. Antibody Titers of Dog Cohort

Overall, protective antibody titers (PATs) against canine parvovirus type 2 (CPV-2), canine distemper virus (CDV), and canine adenovirus type 1 (CAdV-1) were detected in 93.3% (112/120), 79.2% (95/120), and 86.6% (104/120) of the oncology dogs, respectively. Compared with the healthy vaccinated reference cohort from our previous study [12], which demonstrated seroprotection rates of 93.8% (758/808) for CPV-2, 69.4% (561/808) for CDV, and 81.6% (659/808) for CAdV-1, the overall humoral immune coverage in oncology patients was largely preserved, indicating no detrimental impact from the neoplastic condition.

**Figure 1:**
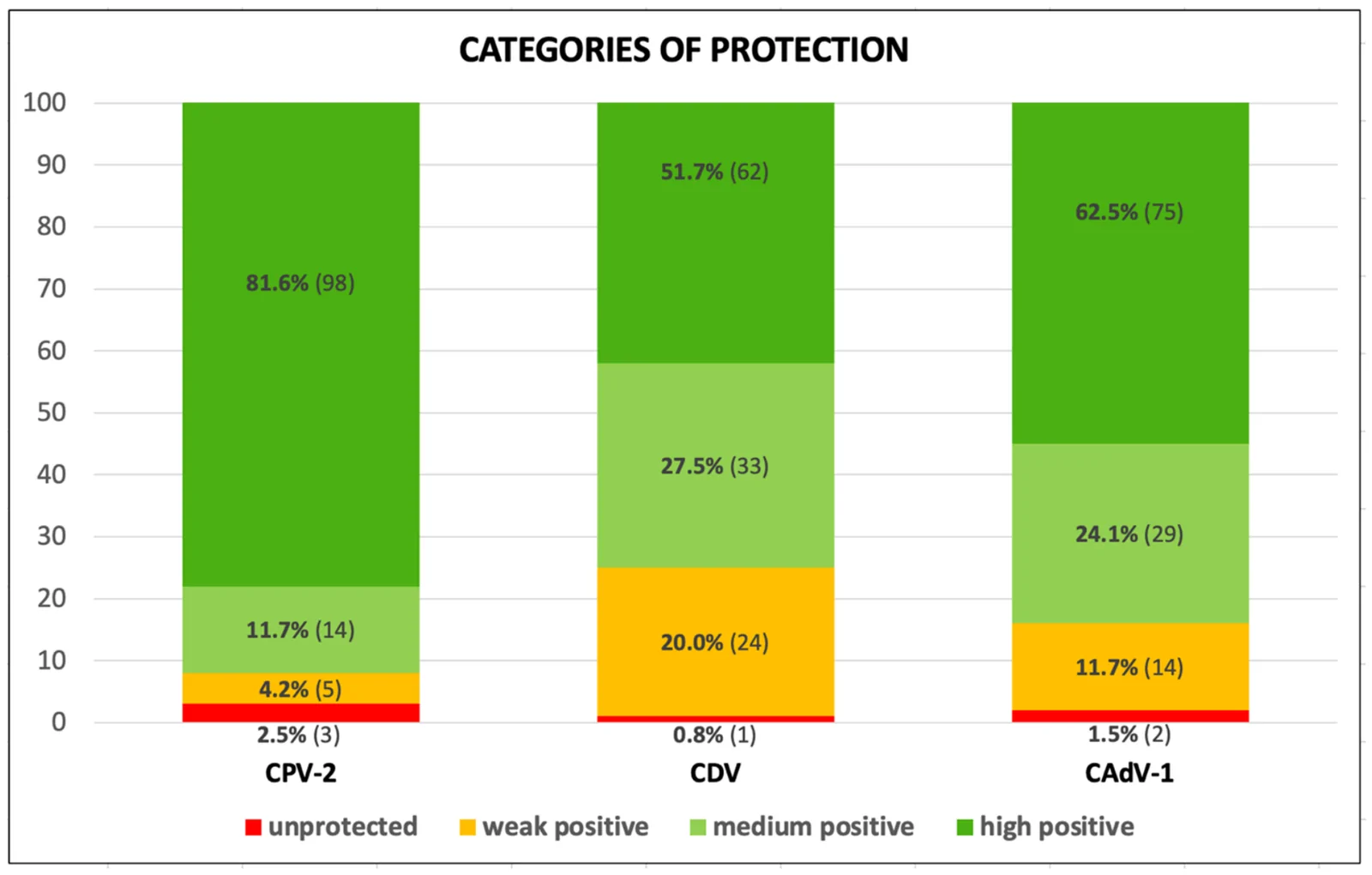
Categories of protection against canine parvovirus type 2 (CPV-2), canine distemper virus (CDV), and canine adenovirus type 1 (CAdV-1) in the 120 oncology canine patients analyzed in this study.

First of all, to provide a comprehensive picture of the humoral protection profile across the entire canine cohort, patients were stratified into four distinct protection categories based on the threshold values of each pathogen: unprotected, weak-positive, medium-positive, and high-positive (Figure 1).

The most robust antibody response was observed for CPV-2, with 81.6% (98/120) of dogs demonstrating high-positive and protective antibody levels and 11.7% (14/120) classified as medium-positive, with the aforementioned overall protection rate of 93.3%. Only a minor fraction fell into the weak-positive (4.2%, 5/120) or unprotected (2.5%, 3/120) categories.

An intermediate pattern was displayed for CAdV-1, with 62.5% (75/120) high-positive and 24.1% (29/120) medium-positive (overall protection rate of 86.6%), while 11.7% (14/120) of patients were weak-positive, and only 1.5% (2/120) unprotected.

CDV showed the lowest protective value: 51.7% (62/120) of dogs showed high-positive results and 27.5% (33/120) medium-positive results, yielding a combined protective rate of 79.2%. These percentages were calculated based on the threshold value recommended by the kit (1:32). However, a notable proportion of dogs (20.0%, 24/120) fell into the weak-positive category, corresponding to a 1:16 titer. This threshold value is widely accepted by reference diagnostic laboratories and in-clinic tests as indicative of clinical protection against infection. Speculatively considering this lower threshold 1:16 (defined by kit specifications as weakly positive but indicating protective humoral immunity), the overall CDV seroprotection rate reached 95.0% (114/120), with only six dogs remaining unprotected.

Furthermore, as expected, antibody levels of the oncology dogs showed significant variability according to dog’s sex, age, breed size, time elapsed from the last core vaccination and tumor type. Since this study aims to evaluate the potential impact of different neoplastic conditions on core vaccine humoral immunity, particular emphasis is placed, in the next section, on analyzing results according to tumor type.

### 3.3. Antibody Titers According to Tumor Type

**Figure 2:**
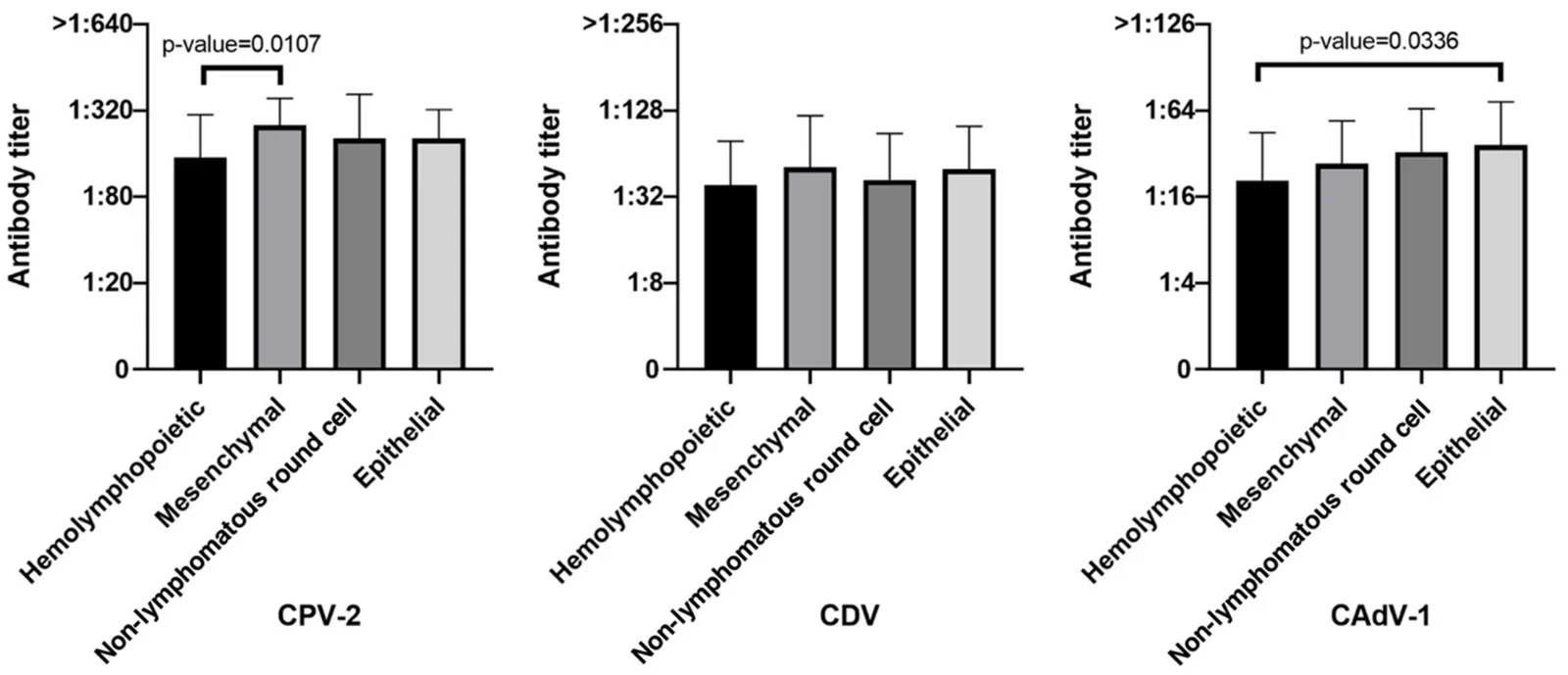
Antibody titers against canine parvovirus type 2 (CPV-2), canine distemper virus (CDV) and canine adenovirus type 1 (CAdV-1) considering the different tumor type affecting the canine cohort (Kruskal–Wallis test).

When comparing the humoral immune response among neoplastic categories, clear variations were observed in mean antibody titers and overall seroprotection rates (Figure 2 and Table 2).

The lowest overall antibody titers and seroprotection levels were recorded in dogs with hemolymphopoietic tumors. This was the most common category in the study (30.8%, 37/120) and comprised neoplasms directly involving the immune system: 33 lymphomas (14 B-cell, 13 T-cell, and 6 unclassified) and 4 leukemias. Within this group, dogs exhibited a significantly lower mean antibody titer for CPV-2 compared to those with mesenchymal neoplasms (*p* = 0.0107), despite maintaining a high overall seroprotection rate (91.9%, 34/37). Similarly, mean antibody titers for CAdV-1 were significantly lower in hemolymphopoietic cases than in epithelial ones (*p* = 0.0336), with an overall seroprotection rate of 83.8% (31/37). For CDV, this group showed the highest proportion of unprotected patients (24.3%, 9/37); when evaluated at the standard 1:32 threshold, PATs were present in 75.7% (28/37) of dogs; however, when lowering the protective threshold to 1:16, the proportion of unprotected dogs dropped sharply to 5.4% (2/37), with PATs reaching 94.6% (35/37).

Conversely, the highest humoral protection was displayed by dogs affected by solid tumors of epithelial origin (16.7%, 20/120), including mammary and non-mammary adenocarcinomas, bladder, renal, and squamous cell carcinomas, as well as those with mesenchymal origin (21.7%, 26/120), encompassing hemangiosarcomas, soft tissue sarcomas, osteosarcomas, chondrosarcomas, and leiomyosarcomas. Both groups achieved 100% seroprotection for CPV-2 (20/20 and 26/26, respectively), while maintaining high protection rates for CDV (90.0% in epithelial tumors and 80.8% in mesenchymal ones) and CAdV-1 (95.0% in epithelial tumors and 88.5% in mesenchymal ones).

Dogs with non-lymphomatous round cell tumors (19.1%, 23/120), which included mast cell tumors and malignant histiocytomas, demonstrated robust humoral immunity closely resembling that of solid epithelial and mesenchymal ones. In this group, seroprotection reached 95.7% (22/23) for both CPV-2 and CAdV-1, and 87.0% (20/23) for CDV.

Finally, the heterogeneous group categorized as “other types” (11.7%, 14/120), which encompassed testicular neoplasms, melanomas, and thymic neoplasms, exhibited lower overall seroprotection rates across all three viruses, showing 71.4% for CPV-2, 57.1% for CDV, and 64.3% for CAdV-1. This finding may be attributed to the small sample size (14) and the heterogeneity of tumors within this group in this category, which likely exert distinct impacts on the immune system. Consequently, this category was excluded from further statistical analysis.

### 3.4. Influence of Individual Features on Antibody Response

To assess whether host characteristics beyond tumor type could affect the humoral immune status, the influence of individual physiological features (sex and reproductive status, age, and breed size) and the time elapsed since the last vaccination on antibody response was analyzed. Regarding these parameters, no statistically significant differences in seroprotection rates were detected across most categories.

**Figure 3:**
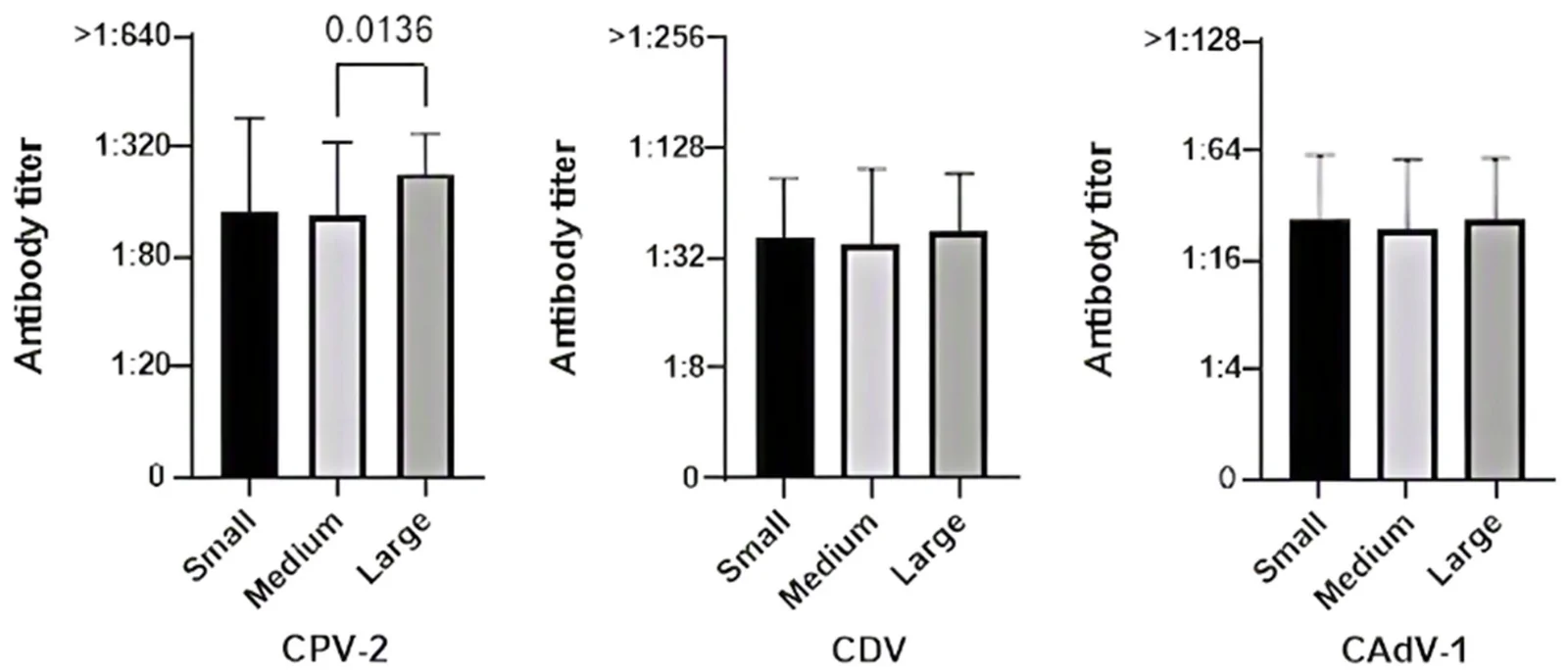
Antibody titers against canine parvovirus type 2 (CPV-2), canine distemper virus (CDV) and canine adenovirus type 1 (CAdV-1) considering the body size of the canine cohort (Kruskal–Wallis test).

The single statistically significant variation regarding body size emerged for CPV-2 (Figure 3). Dogs of medium size (≥10–<25 kg, 39/120) demonstrated a lower rate of protective antibody titers (87.2%, 34/39) compared to large-sized dogs (≥25 kg), which achieved a 100% protection rate (54/54) (*p* = 0.0136). Small-sized dogs (<10 kg, 27/120) maintained a protection rate of 88.9% (24/27) against CPV-2. Breed size did not significantly influence seroprotection for CDV or CAdV-1.

Analysis of the time elapsed since the last core vaccination revealed no statistically significant differences among the groups (<1 year, 43/120; ≥1–<3 years, 56/120, and ≥3 years, 21/120). High protection against CPV-2 persisted across all intervals (93.0% at <1 year, 94.6% at ≥1–<3 years, and 90.5% at ≥3 years). For CDV and CAdV-1, protective titers were detected in 93.0% and 97.7% of dogs vaccinated within <1 year, 85.7% and 92.9% in those vaccinated ≥1–<3 years prior, and 33.3% and 47.6% in dogs vaccinated ≥3 years prior, respectively.

## 4. Discussion

Neoplasia currently represents the primary cause of death in companion animals worldwide, with one in four dogs developing a tumor during their lifetime, particularly beyond 10 years of age [30,31]. In fact, neoplastic incidence rises markedly in older animals, driven by the autonomous, unchecked proliferation of tissues alongside the progressive decline of immune surveillance [32]. According to data from the Veterinary Cancer Society [33] and the Small Animal Veterinary Surveillance Network (SAVSNET) tumor registry, one of the largest open-source databases dedicated to canine and feline oncology [34], the most common malignancies in dogs include lymphoma (accounting for approximately 24% of all new cancer cases), osteosarcoma (an aggressive neoplasm representing 85% of all skeletal tumors), mast cell tumors (the most prevalent cutaneous neoplasm in dogs), oral melanoma, hemangiosarcoma, and transitional cell carcinoma (the most common tumor of the canine urinary tract).

A 2017 Italian study analyzed data from the Piedmont Canine Cancer Registry over a 7.5-year period (2001–2008), identifying 1175 histopathologically confirmed tumors, corresponding to an overall incidence rate of 804 malignant neoplasms per 100,000 dogs per year. The most prevalent malignant subtypes were mast cell tumor, hemangiopericytoma, and mammary gland carcinoma [35]. Similarly, a previous landmark study in Italy [36] evaluated the Animal Tumor Registry of Genoa over an 18-year period (1985–2002), registering 3303 confirmed malignant tumors. This study reported an age-dependent increase in cancer incidence, with a higher rate observed in females compared to males (272.1 vs. 99.3 cases per 100,000 dogs/year). The primary malignancies identified were mammary carcinoma and lymphoma in females, and lymphoma and skin neoplasms in males [36].

Given the high prevalence of these conditions, understanding their impact on host immune defenses is of paramount importance. Clarifying whether vaccine efficacy declines in these patients is critical to prevent their exposure to severe, life-threatening infections. In support of this, some studies have shown that oncology patients frequently exhibit systemic immunosuppression particularly of the cell-mediated compartment, largely mediated by inhibitory factors actively secreted by neoplastic cells [32,37,38,39,40,41,42,43,44].

This study provides a comprehensive evaluation of the antibody response to core vaccinations in cancer-bearing dogs, with emphasis on the potential impact of neoplastic illness on immunologic function and vaccine responsiveness at the time of diagnosis or prior to the initiation of any eventual antineoplastic treatment.

### 4.1. Overall Protective Profile and Comparison with Healthy Reference Cohort

The overall stratification of protection levels (illustrated in Figure 1) offers immediate clinical insight into pathogen-specific humoral resilience in oncology patients. The overwhelming dominance of high-positive titers for CPV-2 (81.6%) underscores the exceptional immunogenicity and persistence of long-term memory plasma cells generated against parvovirus, even in the presence of neoplastic disease.

Regarding CDV, although the initial titer-based classification indicates a higher proportion of weak-positive results (20.0%) compared to CPV-2 (4.2%) and CAdV-1 (11.7%), this result must be interpreted considering diagnostic cut-offs and clinical relevance. While the point-of-care test used in this study (VacciCheck^®^) sets its standard threshold for full protection at a 1:32 CDV titer, extensive literature and gold standard virus neutralization (VN) assays often consider a 1:16 titer sufficient for clinical protection. Speculatively including the weak-positive group in the protected cohort increases the overall CDV protection rate from 79.2% to 95.0%, indicating that the vast majority of canine oncology patients maintain protective immunity against distemper virus despite their underlying disease.

Categorizing patients into these protection levels is particularly valuable for clinical practice: while high- and medium-positive dogs can be safely considered protected without requiring immediate booster vaccinations, dogs falling into the weak-positive or unprotected tiers represent a fragile subset that warrants careful individual risk assessment and targeted monitoring [6,10,11,12,19,22,24,45,46,47]. It must be noted that WSAVA guidelines [6,19] emphasize that any antibody titer detected in an adult animal is an indicator of protection because it denotes the presence of immune memory. It is also true, however, that the same guidelines and many published studies state that antibody titers well correlate with protection; it means that a high titer corresponds to high protection and vice versa.

Our findings show that canine cancer patients maintain a surprisingly robust humoral immunity against core pathogens. These results are fully consistent with—and in fact slightly surpass—the seroprotection rates documented in a previous study conducted by our research group on a broad canine population (*n* = 1027, including 808 healthy vaccinated dogs), which serves as the reference cohort for the present investigation [12]. This confirms that pre-existing humoral immune memory against previously encountered core antigens remains largely preserved despite neoplastic development.

Conversely, a distinct immunological behavior would be expected for novel antigens encountered de novo, as the presence of a tumor and its associated immunosuppressive microenvironment might significantly impair the induction of a primary protective immune response.

A similar persistence of pre-existing immune memory was previously observed by our group in a cohort of canine oncology patients undergoing active chemotherapy [27]. In that investigation, no statistically significant reduction in antibody protection against CPV-2, CDV, or CAdV-1 was detected across various chemotherapeutic protocols, indicating that even cytotoxic treatments do not substantially impair pre-established post-vaccinal immune memory.

To better understand our study population, it is valuable to compare its demographic distribution with recent large-scale epidemiological data from Rafalko et al. [48]. In a cohort of 3452 dogs diagnosed with cancer, the authors evaluated age at diagnosis alongside breed, sex, neuter status, weight, and tumor type to determine the optimal age for initiating cancer screening. While Rafalko et al. focused on establishing optimal screening ages based on breed, body weight, sex, and tumor type, our study specifically evaluated the persistence of protective antibody titers (PATs) against CPV-2, CDV, and CAdV-1 across these demographic and clinical variables. Despite differences in primary study objectives, several notable parallels and distinctions emerge when comparing the two cohorts.

Rafalko’s study population [48] included 2537 purebred dogs (73.5) representing 122 breeds, and 915 mixed-breed (26.5%) dogs; the most represented breeds were Golden Retrievers (16.6%), Labrador Retrievers (15.6%), Boxers (7.0%), Rottweilers (6.6%), and German Shepherds (4.0%). Similarly, in our study, 69 dogs were purebred (57.5%) and 51 were crossbred (42.5%), with the Golden Retriever being the most represented breed (13.0%), followed by Beagle and French Bulldog (7.2%), and by Border Collie, Boxer, German Shepherd and Pinscher (5.8%).

A higher prevalence of neoplasms in purebred dogs has also been reported in previous studies [31,33,35,42,49], alongside breed predispositions to specific tumor types. Consistent with our observations, Golden Retrievers are often the most overrepresented breed, followed by Boxers and German Shepherds—a pattern fully consistent with our cohort [30,31,33,35,49,50,51,52,53].

### 4.2. Immunological Impact of Tumor Types: Hemolymphopoietic vs. Solid Malignancies

In Rafalko’s study [48], the most common cancer types were lymphoma/lymphoid leukemia, followed by osteosarcoma, mast cell tumor, hemangiosarcoma, and soft tissue sarcoma. Hematologic malignancies and mast cell tumors were diagnosed at younger median ages (<8 years) compared to carcinomas and melanomas (11 years). Overall, the median age at diagnosis was 8.8 years (range: <1 to 20 years).

Hemolymphopoietic tumors (particularly lymphomas) constituted the largest diagnostic category in our study as well (*n* = 37/120, 30.8%), followed by mesenchymal (*n* = 26), non-lymphomatous round cell (*n* = 23), and epithelial (*n* = 20) tumors. Importantly, despite the potential immunosuppressive nature of hematologic cancers and their treatments, dogs with hemolymphopoietic malignancies retained high protection rates for all three diseases (91.9% for CPV-2, 75.7% for CDV, and 83.8% for CAdV-1). Although mesenchymal and epithelial tumor groups achieved protection rates of up to 100% for CPV-2, clinically reassuring antibody levels were consistently observed across all major tumor categories.

In the present study, the distribution of oncology diagnoses closely reflected the epidemiological landscape described in the literature, encompassing a wide variety of systemic and localized malignancies, predominantly lymphomas, mast cell tumors, and sarcomas. When analyzing the impact of specific pathological forms, the underlying type of neoplasm did not emerge as a critical confounding factor disrupting humoral immunity, as seroprotection rates remained consistently high across the different neoplastic categories.

When evaluating the influence of specific neoplastic histotypes, a subtle distinction emerged between hemolymphopoietic malignancies (predominantly lymphomas) and solid tumors (such as carcinomas, sarcomas, and mast cell tumors). Given that hemolymphopoietic neoplasms directly involve immune-lineage cells and disrupt lymphoid tissue architecture, a degree of immunological impairment is biologically plausible. Indeed, dogs with hematopoietic tumors displayed slightly lower seroprotection rates or mean antibody titers for CDV and CAdV-1 compared to those with solid tumors.

Crucially, however, this variation was purely quantitative and did not translate into a failure of humoral immunity. The vast majority of dogs with hemolymphopoietic tumors retained protective antibody levels, consistently above the established threshold for seroprotection. In contrast, patients with solid tumors exhibited seroprotective profiles virtually indistinguishable from healthy reference cohort from our previous study, likely reflecting the preservation of non-involved secondary lymphoid organs and bone marrow niches.

Rather than contradicting our central hypothesis, the maintenance of protective immunity even in lymphoproliferative disorders strongly reinforces the concept that established humoral immune memory is remarkably resilient. Because long-lived plasma cells and memory B cells generated prior to oncogenesis reside in protective survival niches, their antibody-secreting capacity remains functional regardless of the anatomical or histological type of neoplasm.

These findings may appear to contrast with literature evidence indicating that hematopoietic malignancies typically induce immune dysregulation via leukocyte inhibition, reduced production of key immune mediators, and suppression of immune cell interactions [32,54,55]. Furthermore, lymphoproliferative disorders are well known to impair adaptive immunity by directly disrupting B- and T-cell dynamics.

A plausible explanation for this apparent contradiction lies in the distinct compartmentalization of adaptive immunity. While active immune dysregulation severely impairs primary immune activation and cell-mediated interactions required for de novo responses, pre-existing humoral immune memory relies predominantly on long-lived plasma cells (LLPCs) that home to specialized bone marrow niches. These fully differentiated cells continuously secrete high-affinity antibodies without requiring ongoing T-cell help or active B-cell receptor stimulation [56,57]. Consequently, even in the presence of marked disruption within the circulating and nodal lymphocyte pools, pre-established serological protection against core pathogens remains surprisingly durable and resilient.

### 4.3. Influence of Individual Features and Vaccination Timelines

Beyond neoplastic disease, individual host characteristics and vaccination history represent well-established determinants of humoral immunity.

Regarding sex and reproductive status, in Rafalko’s cohort [48], males were slightly overrepresented (55%), and intact animals were significantly younger at diagnosis than neutered ones. In our population, females comprised 55% (*n* = 66/120, of which 44 were spayed) and males 45% (*n* = 54/120, of which 19 were castrated). Overall, high rates of seroprotection were observed across all sex/neutering subgroups, with PATs exceeding 89.0% for CPV-2 and remaining high across CDV and CAdV-1, without statistically significant differences in antibody levels. Despite some minor variations, such as slightly higher CPV-2 protection rates in spayed females compared with intact females, and higher CDV and CAdV-1 protection rates in castrated males compared with intact males, these results agree with the literature suggesting that sex hormones do not consistently or decisively influence post-vaccination antibody responses even during neoplastic disease [12].

When considering age and tumor susceptibility, Rafalko et al. [48] reported an overall median age at cancer diagnosis of 8.8 years, with senior and geriatric dogs comprising the vast majority of cases. In our cohort, the majority of subjects were similarly concentrated in the senior (*n* = 78/120, 65%) and geriatric (*n* = 25/120, 20.8%) groups. Crucially, our findings demonstrate that high levels of protective immunity (particularly against CPV-2) are robustly maintained across all age categories, only with slightly lower seroprotection rates recorded in older oncology patients. This finding is best explained by physiological immunosenescence, characterized by the age-related decline of adaptive immune function and altered plasma cell niche dynamics, rather than a direct immunosuppressive effect exerted by the neoplastic tissue [4,20,21,58,59]. It is also noteworthy that in the cohort analyzed by Rafalko et al. [48], pediatric cases (<1 year of age) represented only 0.4% (*n* = 14) of cancer diagnoses. Similarly, our study included no patients under one year of age, as all subjects belonged to adult, senior, or geriatric categories, a finding that further underscores the strongly age-dependent risk of oncogenesis in the canine population.

When evaluating breed size and body weight, Rafalko et al. [48] identified a strong inverse relationship between body weight and age at cancer diagnosis, with large and giant breeds diagnosed at significantly younger ages. In our study, large dogs (>25 kg) represented the largest subgroup (*n* = 54/120, 45.0%) that, interestingly, maintained exemplary seroprotection. Statistical analysis revealed a significant difference in CPV-2 seroprotection across breed sizes, with large dogs exhibiting significantly higher protection rates than medium-sized dogs (*p* = 0.0136), alongside a similar non-significant trend when compared to small dogs. This finding diverges from established literature reporting that smaller dogs often show higher responsiveness to antigenic stimulation and vaccination than larger counterparts. Nevertheless, this observation is fully consistent with previous findings from our group involving a broader cohort of 1027 dogs [12] and a separate investigation on 350 senior and geriatric dogs [21], both of which demonstrated that large-breed dogs possess significantly higher CPV-2 antibody titers than medium and small dogs. A plausible explanation—though speculative—is that large dogs often spend more time outdoors, engage in more frequent physical activity, and have greater interaction with high-density canine environments (e.g., dog parks) [60,61,62,63]. Given the high environmental persistence of CPV-2 [64,65,66,67,68,69], this lifestyle could facilitate subclinical exposure to the virus, acting as a natural immune boost and helping to sustain high antibody titers long after vaccination. However, since environmental exposure and lifestyle factors were not directly measured in the present study, this hypothesis must be interpreted cautiously. Nevertheless, whether titers are maintained solely through long-lived vaccine memory or bolstered by environmental re-exposure, these findings confirm that the host’s immune system retains the capacity to mount and preserve protective humoral memory despite the presence of underlying neoplastic disease.

Finally, evaluating the time elapsed since the last core vaccination revealed a progressive decline in seroprotection rates for CDV and CAdV-1 in dogs vaccinated >3 years prior, whereas antibody protection against CPV-2 remained exceptionally high and stable regardless of the time interval. Of the 21 dogs that had been vaccinated more than three years ago, 14 were senior (66.7%), 4 were geriatric (19.0%) and only 3 were adult (14.3%). In addition to the longer interval since the last core vaccination, the advanced age of these patients may also have contributed to this finding. Crucially, this pathogen-specific kinetic profile closely mirrors the pattern routinely observed in healthy canine populations [12]. This demonstrates that the gradual drop in CDV and CAdV-1 antibody titers reflects the physiological waning of circulating immunoglobulins over time, rather than a tumor-induced impairment of established immune memory.

In summary, our findings indicate that pre-existing core vaccine antibody titers are largely preserved in treatment-naïve canine oncology patients. Although these results rely on a descriptive comparison with an external historical reference cohort rather than a prospectively matched control group, the high prevalence of protective titers suggests that underlying malignancy does not cause a decline of specific core vaccine humoral immunity. From a practical perspective, this provides valuable reassurance to veterinary clinicians and pet owners when evaluating vaccination schedules at the time of cancer diagnosis.

## 5. Conclusions

In dogs diagnosed with malignant neoplasms, evaluating protective immunity against core vaccine-preventable diseases, namely canine parvovirus (CPV-2), canine distemper virus (CDV), and canine adenovirus type 1 (CAdV-1), is supported by several compelling rationales. In an oncology patient whose general health may already be compromised, even a minor infectious disease could yield severe, life-threatening consequences. Ensuring adequate protection against major core pathogens is therefore paramount in these vulnerable animals. Conversely, when managing a patient with a malignant tumor who will likely undergo intensive surgical and medical treatments, the veterinary clinician must avoid administering unnecessary interventions, including non-essential booster vaccinations [11].

This study demonstrates that seroprotection against CPV-2, CDV, and CAdV-1 remains high in the vast majority of vaccinated oncology patients prior to starting antineoplastic therapy, as confirmed by the in-clinic VacciCheck^®^ titer test. This indicates that immunological memory is not compromised by the presence of a neoplasm, even in senior and geriatric patients naturally subject to immunosenescence, thereby confirming findings from previous studies stressing that old dogs can die from a core vaccine-preventable disease especially if unvaccinated or inadequately vaccinated [5,6,12,21,23,59]. As anticipated, the lowest antibody titers, though still above the protective threshold, were detected in dogs with hemolymphopoietic tumors (particularly lymphomas).

Furthermore, a previous study by our group evaluating dogs vaccinated against CPV-2, CDV, and CAdV-1 undergoing chemotherapy revealed no statistically significant variations in antibody protection across any of the chemotherapy protocols investigated. These findings suggest that, contrary to common expectations, chemotherapy does not exert a marked immunosuppressive effect on post-vaccinal antibody responses. Thus, immunological memory toward previously encountered antigens is preserved over time, providing long-lasting protection despite the presence of neoplastic disease and its associated treatments [27].

Among available in-clinic rapid assays, VacciCheck^®^ was selected for its semi-quantitative dot-ELISA technology, which provides superior diagnostic performance compared to immunochromatographic tests (mostly qualitative). Widely regarded as the point-of-care reference standard for core vaccine antibody titration, this assay is officially approved by international regulatory bodies and extensively validated against laboratory reference methods (VN and HI) [11,70,71,72], ensuring high diagnostic reliability and direct clinical applicability.

The use of rapid point-of-care tests to assess core antibody protection is also recommended by the recent AAHA Canine Vaccination Guidelines to determine the true immune status of individual oncology patients: a positive result indicates that the animal is protected and revaccination is unnecessary; if the result is negative, the decision to revaccinate remains at the veterinarian’s discretion based on the patient’s clinical condition and lifestyle [10].

These results, albeit preliminary, provide valuable insights for veterinary clinicians to refine the clinical management of canine cancer patients by highlighting that core vaccine boosters may not represent an immediate priority. Crucially, these findings are also particularly useful in reassuring owners who, when faced with a diagnosis of malignant neoplasia, are often deeply concerned about their pet’s quality of life.

While this study provides valuable clinical insights into the persistence of core vaccine antibodies in canine cancer patients, some limitations should be acknowledged.

First, although the overall sample size (*n* = 120 dogs) was sufficient to identify statistically significant trends, the distribution across specific subgroups remains a constraint. Specifically, certain tumor categories, such as epithelial neoplasms (*n* = 20) and less frequent histotypes (*n* = 14), as well as specific demographic classes, were represented by relatively small numbers of subjects. This subgroup fragmentation may limit the statistical power required to detect subtle variations across rare neoplastic entities or specific clinical stages.

Another limitation is that advanced clinical stage, metastatic status, and compromised nutritional status were not systematically recorded or analyzed as independent variables affecting antibody titers. While severe tumor progression or malnutrition could theoretically exert a more pronounced systemic immunosuppressive effect, this is unlikely to have significantly impacted our main results, as the primary objective of this study was to evaluate the overall presence of PATs against core pathogens in a real-world clinical cohort.

Similarly, underlying non-neoplastic chronic comorbidities (e.g., chronic renal disease, endocrinopathies, or systemic inflammatory states) were not systematically controlled as independent variables in the analysis and might have contributed to individual variation in antibody levels. Nevertheless, the high prevalence of PATs observed across the cohort indicates that pre-existing humoral memory remains remarkably resilient even in the presence of potential concurrent comorbidities.

Additionally, the study utilized a cross-sectional design with a single time-point assessment of antibody titers upon diagnosis, prior to treatment. Consequently, longitudinal tracking of antibody kinetics over time in individual patients was not performed. Furthermore, immune evaluation was restricted to humoral immunity via serum/plasma antibody titers; cell-mediated immune responses, which also play a fundamental role in antiviral protection and tumor surveillance, were not assessed.

Furthermore, seroprotection was evaluated using the in-clinic semiquantitative VacciCheck^®^ assay rather than gold-standard laboratory procedures (such as serum neutralization or hemagglutination inhibition). While gold-standard assays directly measure functional antibody activity (e.g., neutralization or hemagglutination inhibition), the dot-ELISA assay detects total antigen-binding specific IgG levels as a surrogate marker. Nevertheless, this rapid test is fully validated against classical reference methods: the detected antibody titers correlate reliably with clinical protection, providing the foundation for its strong endorsement by international guidelines (e.g., WSAVA [6,19] and AAHA [10]) and reflecting real-world veterinary practice.

Finally, the study population was geographically localized, which might slightly affect generalizability due to regional variations in breed popularity, pathogen pressure, and core vaccination protocols. Additional potential confounding variables, such as underlying non-neoplastic comorbidities, subtle differences in nutritional status, or specific vaccine brands, were not fully controlled and could have exerted a minor influence on individual antibody levels.

Despite these limitations, dogs diagnosed with malignancy maintain a high prevalence of PATs against CPV-2, CDV, and CAdV-1, with no clear evidence of major humoral impairment compared to historical healthy reference data. These findings suggest that routine core vaccine boosters may not represent an immediate priority in canine cancer patients at presentation, thereby reassuring veterinary clinicians and pet owners and allowing the clinical focus to remain on oncological staging and treatment planning.

## Figures and Tables

**Table 1:** Tumor types affecting the dogs of this study considering their histogenic origin and examples for each category.

Category of Tumors	% (*n*. of Cases)	Type of Tumors
Hemolymphopoietic	30.8 (37)	Lymphoma (B, T, unclassified) Leukemia
Mesenchymal	21.7 (26)	Hemangiosarcoma (splenic, pericardial, hepatic) Soft tissue sarcoma Osteosarcoma Chondrosarcoma Leiomyosarcoma
Non-lymphomatous round cell	19.1 (23)	Mastocytoma (mast cell tumors) Malignant histiocytoma
Epithelial	16.7 (20)	Adenocarcinoma (mammary and non-mammary) Carcinoma (bladder, squamous cell, renal)
Other types	11.7 (14)	Testicular neoplasm Melanoma Thymic neoplasm

**Table 2:** Percentages and numbers (*in italics* in brackets) of 120 oncology dogs with protective and unprotective results for canine parvovirus type 2 (CPV-2), canine distemper virus (CDV), and canine adenovirus type 1 (CAdV-1) according to sex and reproductive status, age, size, type of tumor and time elapsed since the last core vaccination.

	Protective Antibody Titers (PATs) * % (*n. of Dogs*)			Unprotective Antibody Titers % (*n. of Dogs*)		
	CPV-2	CDV	CAdV-1	CPV-2	CDV	CAdV-1
**OVERALL VALUE**	**93.3** (*112*/*120*)	**79.2** (*95*/*120*)	**86.6** *(104*/*120*)	**6.7** (*8*/*120*)	**20.8** *(25*/*120*)	**13.2** (*16*/*120*)
**Sex and reproductive status**						
Intact females (*22*/*120*)	**90.9** (*20*/*22*)	**77.3** (*17*/*22*)	**90.9** (*20*/*22*)	**9.1** (*2*/*22*)	**22.7** (*5*/*22*)	**9.1** (*2*/*22*)
Neutered females (*44*/*120*)	**93.2** (*41*/*44*)	**77.3** (*34*/*44*)	**81.8** (*36*/*44*)	**6.8** (*3*/*44*)	**22.7** (*10*/*44*)	**18.2** (*8*/*44*)
Intact males (*35*/*120*)	**97.1** (*34*/*35*)	**80.0** (*28*/*35*)	**85.7** (*30*/*35*)	**2.9** (*1*/*35*)	**20.0** (*7*/*35*)	**14.3** (*5*/*35*)
Neutered males (*19*/*120*)	**89.5** (*17*/*19*)	**84.2** (*16*/*19*)	**94.7** (*18*/*19*)	**10.5** (*2*/*19*)	**15.8** (*3*/*19*)	**5.3** (*1*/*19*)
**Age**						
Adults (*17*/*120*)	**100.0** (*17*/*17*)	**82.4** (*14*/*17*)	**88.2** (*15*/*17*)	**0.0** (*0*/*17*)	**17.6** (*3*/*17*)	**11.8** (*2*/*17*)
Seniors (*78*/*120*)	**91.0** (*71*/*78*)	**75.6** (*59*/*78*)	**85.9** (*67*/*78*)	**9.0** (*7*/*78*)	**24.4** (*19*/*78*)	**14.1** (*9*/*78*)
Geriatrics (*25*/*120*)	**96.0** (*24*/*25*)	**88.0** (*22*/*25*)	**88.0** (*22*/*25*)	**4.0** (*1*/*25*)	**12.0** (*3*/*25*)	**12.0** (*3*/*25*)
**Size**						
Small (<10 kg) (*27*/*120*)	**88.9** (*24*/*27*)	**74.0** (*20*/*27*)	**88.9** (*24*/*27*)	**11.1** (*3*/*27*)	**26.0** (*7*/*27*)	**11.1** (*3*/*27*)
Medium (≥10–<25 kg) (*39*/*120*)	**87.2** (*34*/*39*)	**71.8** (*28*/*39*)	**84.6** (*33*/*39*)	**12.8** (*5*/*39*)	**28.2** (*11*/*39*)	**15.4** (*6*/*39*)
Large (≥25 kg) (*54*/*120*)	**100.0** (*54*/*54*)	**87.0** (*47*/*54*)	**87.0** (*47*/*54*)	**0.0** (*0*/*54*)	**13.0** (*7*/*54*)	**13.0** (*7*/*54*)
**Type of tumor**						
Hemolymphopoietic (*37*/*120*)	**91.9** (*34*/*37*)	**75.7** (*28*/*37*)	**83.8** (*31*/*37*)	**8.1** (*3*/*37*)	**24.3** (*9*/*37*)	**16.2** (*6*/*37*)
Mesenchymal (*26*/*120*)	**100.0** (*26*/*26*)	**80.8** (*21*/*26*)	**88.5** (*23*/*26*)	**0.0** (*0*/*26*)	**19.2** (*5*/*26*)	**11.5** (*3*/*26*)
Non-lymph. round cell (*23*/*120*)	**95.7** (*22*/*23*)	**87.0** (*20*/*23*)	**95.7** (*22*/*23*)	**4.3** (*1*/*23*)	**13.0** (*3*/*23*)	**4.3** (*1*/*23*)
Epithelial (*20*/*120*)	**100.0** (*20*/*20*)	**90.0** (*18*/*20*)	**95.0** (*19*/*20*)	**0.0** (*0*/*20*)	**10.0** (*2*/*20*)	**5.0** (*1*/*20*)
Other types (*14*/*120*)	**71.4** (*10*/*14*)	**57.1** (*8*/*14*)	**64.3** (*9*/*14*)	**28.6** (*4*/*14*)	**42.9** (*6*/*14*)	**35.7** (*5*/*14*)
**Time after last core vaccination**						
<1 year (*43*/*120*)	**93.0** (*40*/*43*)	**93.0** (*40*/*43*)	**97.7** (*42*/*43*)	**7.0** (*3*/*43*)	**7.0** (*3*/*43*)	**2.3** (*1*/*43*)
≥1–<3 years (*56*/*120*)	**94.6** (*53*/*56*)	**85.7** (*48*/*56*)	**92.9** (*52*/*56*)	**5.4** (*3*/*56*)	**14.3** (*8*/*56*)	**7.1** (*4*/*56*)
≥3 years (*21*/*120*)	**90.5** (*19*/*21*)	**33.3** (*7*/*21*)	**47.6** (*10*/*21*)	**9.5** (*2*/*21*)	**66.7** (*14*/*21*)	**52.4** (*11*/*21*)

* An antibody titer is considered protective if its value is equal to or higher than the threshold value for the specific disease (1:80 for CPV-2, 1:32 for CDV and 1:16 for CAdV-1).

## Data Availability

The authors confirm that the datasets analyzed during the study are available from the first author or the corresponding author upon reasonable request.

## Appendix A

**Table A1:** VacciCheck^®^: correspondence between S scale units and antibody titers, sensitivity, and specificity for canine parvovirus type 2 (CPV-2), canine distemper virus (CDV), and canine adenovirus type 1 (CAdV-1).

	CPV-2 (%)	CDV (%)	CAdV-1 (%)
S0	<1:20	<1:8	<1:4
S1	1:20	1:8	1:4
S2	1:40	1:16	1:8
**S3 (threshold)**	**1:80**	**1:32**	**1:16**
S4	1:160	1:64	1:32
S5	1:320	1:128	1:64
S6	1:640	1:256	1:128
>S6	>1:640	>1:256	>1:128
*Sensitivity (%)*	*88*	*100*	*94*
*Specificity (%)*	*100*	*92*	*93*
